# Identifying the Biological Characteristics Associated with Oviposition Behavior of Tea Leafhopper *Empoasca onukii* Matsuda Using the Blue Light Detection Method

**DOI:** 10.3390/insects11100707

**Published:** 2020-10-16

**Authors:** Qi Yao, Huining Zhang, Long Jiao, Xiaoming Cai, Manqun Wang, Zongmao Chen

**Affiliations:** 1 Tea Research Institute, Chinese Academy of Agricultural Sciences, Hangzhou 310008, China; 269yao103@163.com (Q.Y.); zhanghuining2010@sina.com (H.Z.); jiaolong@tricaas.com (L.J.); cxm_d@tricaas.com (X.C.); 2 College of Plant Science and Technology, Huazhong Agricultural University, Wuhan 430070, China

**Keywords:** *E. onukii*, blue light detection method, oviposition behavior, egg detection, intact tea shoots

## Abstract

**Simple Summary:**

The tea leafhopper (*Empoasca onukii* Matsuda) is currently one of the most threatening pests in tea gardens in China. To locate and count tea leafhopper eggs, stereomicroscopy is a conventional method by dissecting the tender tissues, which is both time- and labor-consuming. The scarcity of a verified method to directly observe and investigate intact eggs within tea shoots impedes the research efforts to test the oviposition behavior of *E. onukii*. Herein, comparing against the stereomicroscope detection method (SMDM), we evaluated the blue light detection method (BLDM), a technique recently developed for other species in detecting *E. onukii* eggs directly and non-destructively within the tender shoot for four tea cultivars. The conclusion indicated that BLDM could correctly measure the egg laying quantity of *E. onukii* on intact tea shoots, and its accuracy was not affected by both tea cultivars and egg density in the tender shoot. Furthermore, the biological characteristics concerning the oviposition behaviors that have rarely been reported previously for *E. onukii* were investigated using the BLDM. Our findings provide insights for the basic research and theoretical evidence, and allow the follow-up studies for the strategy and mechanism associated with the egg laying behavior of *E. onukii*.

**Abstract:**

Tea leafhopper (*Empoasca onukii* Matsuda) is amongst the key pests in tea plantations around the East Asian region. Stereomicroscopy is a conventional method used for detecting tea leafhopper eggs by dissecting the tender tissues. However, there is a need for a faster and more efficient method to directly observe and investigate intact eggs within tea shoots. The absence of a proven method limits research efforts for determining the oviposition behavior of *E. onukii*. Herein, we applied the blue light detection method (BLDM), a technique recently developed for other species, in order to detect *E. onukii* eggs directly and non-destructively within the tender shoot. In addition, we compared BLDM against the traditional stereomicroscope detection method (SMDM) for four tea cultivars. Notably, our results revealed that BLDM was precise and effective in measuring the egg laying quantity of *E. onukii* on intact tea shoots. Neither tea cultivars nor egg density in the tender shoot significantly affected the accuracy of BLDM. Furthermore, biological characteristics that have rarely been reported previously for *E. onukii* were investigated using the BLDM, including zygote duration, ovipositional rhythm, egg distribution within the tender shoot, and in different leaf positions, numbers of eggs laid by a single female daily, and laid by the entire generation. Therefore, these findings provide insights into the basic and theoretical evidence for the strategy and mechanism associated with the oviposition behavior of *E. onukii*.

## 1. Introduction

Oviposition behavior is important among many insect taxa for ontogeny, reproduction, and maintaining their population in an ecosystem. The dynamism of egg laying in different insect species has application to their life history, behavioral habits, reproductive strategies, and evolutionary patterns, ultimately with both theoretical and practical value. Thus, the study of oviposition behavior provides a better understanding of bionomics for agricultural and forestry pests, and can ultimately contribute to a more effective and sustainable pest prevention and management approaches [1,2]. Amongst the tea leafhoppers, *Empoasca onukii* Matsuda (Hemiptera, Cicadellidae) is the dominant pest species, widely distributed in major tea-producing plantations in China and Japan [3,4]. The adults and nymphs damage the tender tea shoots by piercing into and sucking sap leading to “hopperburn” symptoms on infested shoots of tea plants [5]. Adult female *E. onukii* often insert their eggs into the epidermis of tender stems, also causing hopperburn symptoms that, when combined with feeding damage, leads to a 50–70% yield reduction [6].

The majority of previous studies on the egg laying ecology of tea leafhopper focused on their egg distribution. For instance, in a study by Kosugi (1996), the abdomen of an overwintering female was dissected, targeting mature ova, to compare their oviposition both in the laboratory and in the open field. The results demonstrated that *E. onukii* in Shizuoka Prefecture of Japan annually oviposited beginning in late March [7]. New tea shoots dissected and assessed for eggs under a microscope showed that *E. onukii* females preferred laying eggs on the second and third leaf positions of a tender stem [8]. Similarly, a study by Wang et al. (2008) on the Yunnan large-leaf species in Yingde, Guangdong province, China, demonstrated that 79.64% of eggs were distributed in the second and third leaf positions of tender shoots [9]. Moreover, a study investigating tea leafhopper eggs in organic tea gardens in Fujian, China, revealed that 32.07 and 27.01% eggs oviposited in the second and third leaf positions, respectively, of tender shoots on Tieguanyin (oolong tea) cultivar. Notably, there were 35.42 and 28.81% of eggs oviposited in the second and first leaf positions, respectively, in the Fuding White variety [10]. Furthermore, the use of *Trifolium repens*, *Medicago hispida*, *Calendula officinalis*, and other natural weeds as intercrops in tea plantations has had a significant statistical effect on the egg laying of *E. onukii* on different leaf positions of tea shoots. Most eggs were deposited along the second to fifth leaf position of the tender shoots, and all of them showed the highest numbers at the fourth leaf position [11].

Tea leafhopper oviposition behavior is highly affected by environmental factors. For example, shading can significantly increase egg density in fresh tea shoots, whereas the fecundity of *E. onukii* female adults declined along with the gradual increase or decrease in optimal temperature, beginning at 23–25 °C [12,13,14]. Moreover, the egg laying preference of the tea leafhopper also can be influenced by tea varieties, the topography of tea gardens (e.g., high-mountain versus flat-land), and intercrops in tea plantations such as *Trifolium repens* and *Vigna sinensis* [15,16,17]. Previous studies have mostly focused on a comparison of egg distribution in the different leaf positions of tea garden tender shoots, with differences attributed to various causative factors [18,19]. The narrow focus of previous studies is due to only limited information on the oviposition process and egg laying rate of females during their life spans. Most studies based their statistical comparisons by counting *E. onukii* eggs within dissected tea shoots by the use of a stereomicroscope. Unfortunately, this technique is both time- and labor-consuming, with challenges for precision [20]. Therefore, it has become imperative to identify and evaluate more effective technology, which can directly and accurately investigate the eggs of tea leafhoppers on tender shoots, as well as the behavioral ecology related to oviposition.

Currently, there is no verified method that investigates tea leafhopper eggs on undissected shoots. Boll and Herrmann (2001) reported a simplified method to find the eggs of *E. vitis* in grape leaves. Blue light, illuminating the grape leaves, is passed through a pair of special glasses (blue light filter), the embedded eggs then appear as a green spot that is visible to the naked eye [21,22]. Like *E. vitis*, *E. onukii* females mainly deposit their eggs in the tender tissues of stems rather than leaves; however, whether tender stem thickness impacts the statistical accuracy of the blue light detection method (BLDM) is unknown. Additionally, varying colors of the epidermis of tender shoots on tea cultivars must also be considered during egg detection. Therefore, in our study, the feasibility of using BLDM was evaluated, and compared with the stereomicroscope dissections in investigating *E. onukii* eggs on the intact tea shoots of four tea cultivars. Biological characteristics associated with ovipositional behavior were studied to comprehensively determine the egg laying bionomics in *E. onukii*.

## 2. Materials and Methods

### 2.1. Insects and Tea Shoots

The adults of *E. onukii* were collected by sweepnet in Longjing 43 tea plantations at the Tea Research Institute, Chinese Academy of Agricultural Sciences, in Hangzhou, Zhejiang province, China (30.18°N, 120.09°E). Insects were reared under laboratory conditions at temperatures of 25 ± 2 °C, relative humidity of 70 ± 3%, photoperiod of 14L:10D, and with hydroponic tea tips collected from the same tea gardens. The tea cultivars for evaluations were Longjing 43, Maoxie, Huangjinya, and Zijuan cultivated in National Tea Germplasm Resources Garden, Hangzhou, China. A brief summary of varietal characteristics is indicated in Table 1. The tender shoots of 5 year-old tea bushes were excised such that at least 8 leaf positions were available.

### 2.2. The Accuracy of Blue Light Detection Method (BLDM) for Locating Eggs of E. onukii within Intact Tea Shoots

#### 2.2.1. Observation and Location of *E. onukii* Eggs by BLDM Inside Intact Tea Shoots

**Figure 1:**
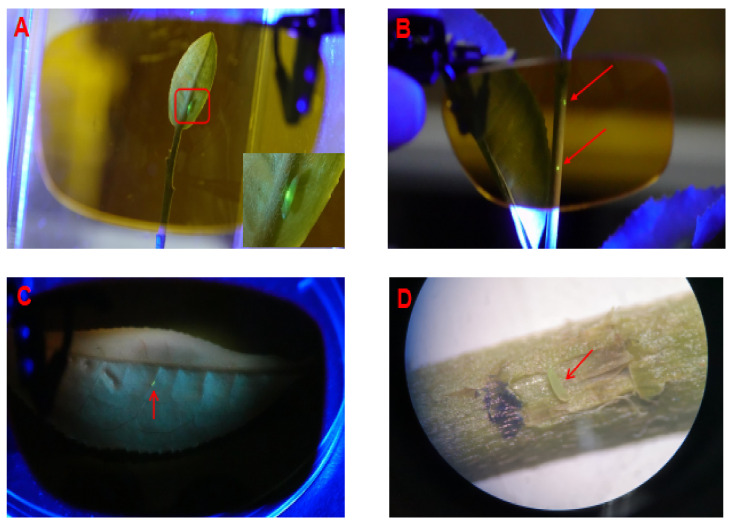
Images of *E. onukii* eggs under the blue light detection method (BLDM): (**A**) gravid female (enlarged size of *E.onukii* female laying on the lower right corner); (**B**) eggs on the tender stem; (**C**) egg on the leaf vein; and (**D**) an egg detected by stereomicroscope.

Currently, there are no reports of a method to directly detect *E. onukii* eggs within intact tea shoot, except for dissecting tender stem and checking under the stereomicroscope. We therefore evaluated the blue light detection method originally developed by Herrmann and Boll (2004), using a blue light source of 460 nm wavelength, 200 wattage, and the filter glasses (PTK, Germany). Since we successfully detected *E. onukii* eggs inside intact stems and leaves (Figure 1), we adopted this methodology for subsequent experiments.

#### 2.2.2. Accuracy Examination of BLDM for Investigating Eggs of *E. onukii*

The accuracy of BLDM in detecting *E. onukii* eggs in intact tender tissues was verified on Longjing 43, Maoxie, Huangjinya, and Zijuan tea cultivars. The initial investigation suggested that blue light was unable to visualize *E. onukii* eggs oviposited deeply in stem epidermis. It was also uncertain whether all green fluorescent points observed were *E. onukii* eggs. Therefore, the tea shoots of each cultivar were divided into two groups of green and no-green dots after BLDM checking; each group had 10 tea shoots. Thereafter, the shoots of each group were inserted into moistened floral foams, which were then placed into a PMMA (polymethyl methacrylate) Insect Cage (20 cm × 20 cm × 20 cm) made of a transparent acrylic board with gauze doors on both sides for ventilation. The hatching period for *E. onukii* eggs is approximately 7 d under an indoor temperature of 25 °C [23]. Therefore, floral foams were observed daily for hatched nymphs for 7 d, by which day most eggs would have hatched. Each day before day 7, tender stems and leaves were dissected for unhatched eggs and viewed under the stereomicroscope (PXS-D, Shanghai Sixth Factory for Optical Instruments) for confirmatory analysis.

#### 2.2.3. Comparison of BLDM and SMDM in the Detection of *E. onukii* Eggs on High-Egg-Density Tea Shoot

We compared the accuracy of the traditional stereomicroscope detection method (SMDM) versus the accuracy of BLDM in detecting the eggs of *E. onukii* under a high-egg-density situation (i.e., more than twice as many eggs as normally laid in the field). For each cultivar, 10 tea shoots were collected from the field and exposed to 200 *E. onukii* adults for 24 h in the laboratory before the shoots were first investigated by blue light, then again by the stereomicroscope method for dissecting shoots. The whole experiment was repeated 3 times and a total of 120 tea shoots of four cultivars were examined.

### 2.3. Biological Characteristics Associated with the Oviposition Behavior of E. onukii

#### 2.3.1. Zygote State

Newly hatched nymphs of *E. onukii* were separated into males and females. After that, the mature females were introduced into a long, narrow glass tube containing a moistened floral foam holding hydroponically-reared, tender tea shoots, as used for breeding. The glass tube was closed with gauze fastened with a rubber band, for ventilation. During the mating of the females and males in the tube, the duration of mating was recorded before the females were transferred to another tube with tender tea shoots. The presence of eggs in females and the evidence of eggs in the tender stem and leaf was detected using the BLDM at 8:30, 13:30, and 20:00 daily.

#### 2.3.2. Ovipositional Rhythm and Egg Distribution

Eight time intervals within 24 h were set in this experiment at 11:30~14:30, 14:30~17:30, 17:30~20:30, 20:30~23:30, 23:30~2:30, 2:30~5:30, 5:30~8:30, and 8:30~11:30. Leafhopper adults were captured in tea gardens during their peak occurrence and reared indoors for 1 d (25 ± 20 °C, 70 ± 3% HR, 14L: 10D). Thereafter, about randomly selected 60 leafhoppers were introduced into the PMMA Insect Cage containing a moistened floral foam, set up in advance with 3 tender tea shoots without eggs. At 3 h intervals, the tea shoots were removed to observe the *E. onukii* eggs deposited in every tender stem and leaf using BLDM. After that, 3 fresh tea shoots were replaced in the PMMA Insect Cages. Statistical comparisons of differences in the timing of the egg laying process were calculated for different periods within 24 h and among different leaf positions on the tender stem. The experiment was repeated four times.

#### 2.3.3. Number of Eggs Laid Daily by a Single Female

Male and female adults of *E. onukii* (10 each) were introduced into a PMMA cage (20 cm × 20 cm × 20 cm). To ensure sufficient egg laying space for the females, 3 clean tender tea shoots without eggs were inserted into moistened floral foam and placed in the cage. Thereafter, tea shoots were removed daily before counting *E. onukii* eggs directly using the BLDM. Fresh tea shoots with no eggs were simultaneously placed in the cage. The assessment duration was 7 days to determine the egg laying rate per day in females. The experiment was repeated four times.

#### 2.3.4. Total Eggs Laid by a Single Female

Sexually immature males and females of *E. onukii* (5 each) were introduced into the PMMA cage with 3 tender tea shoots containing no eggs. Tea shoots were removed after 5 d to count the *E. onukii* eggs using BLDM; simultaneously, fresh tea shoots were added. Thereafter, the assessment continued at 3 day intervals until no eggs were detected or all the adults in the cages had died. The experiment was repeated four times to determine the average number of eggs laid by a single female.

### 2.4. Data Analysis

Statistical software Excel and SPSS 25.0 were used for the data summary and analysis. In all experiments, ANOVA was initially performed. Thereafter, pairwise comparison tests of BLDM accuracy were performed using the least significant difference (LSD) method. In investigations of ovipositional rhythm, the numbers of eggs being oviposited in different periods and distributing in different leaf positions were both compared by the LSD method. Charts were drawn using GraphPad Prism 8.0 using the mean ± SE.

## 3. Results

### 3.1. Video Observation of Egg Laying Behavior of E. onukii

Observations of the egg laying videos recorded in the laboratory clearly indicated that gravid females of *E. onukii* can oviposit inside all parts of the tender shoots, including stem, leaf petiole, and vein. Each adult female identified a suitable site to oviposit. Thereafter, the female held her tarsi immobile, and tilted her body upward by contracting her abdomen. Subsequently, she protruded her ovipositor and inserted it vertically into the epidermis between the propodium and metapedes; after some time, she retracted the ovipositor and finally returned it into her body. The whole process lasted for 2–3 min.

### 3.2. Observation and Clarification of E. onukii Egg by Blue Light Detection Method (BLDM)

Blue light detection method (BLDM) was used to observe the *E. onukii* gravid female (Figure 1A) and its egg morphogenesis inside the tender tissues on the stem and leaf vein (Figure 1B,C). The green dots observed under blue light were marked and verified as *E. onukii* eggs through a binocular stereomicroscope, by the subsequent dissection of the tender tea shoots (Figure 1D). In particular, eggs being deposited in the petiole, leaf vein, and higher leaf area were visible, whereas those oviposited in the lower leaf area (e.g., bud, the first to third leaf position) were invisible, due to weak fluorescence through the eggs embedded deeply in the stem.

### 3.3. Inspection Accuracy of BLDM for E. onukii Eggs

After 7 d, neither eggs nor hatched nymphs were found in the tea shoot groups that started with no eggs, for all four varieties (Longjing 43, Maoxie, Huangjinya, and Zijuan). This result indicated that no extra egg oviposition occurred deep in the tender tissues that could not be detected by blue light. Thus, the thickness of the tender stems of tea shoots did not reduce the accuracy of BLDM in detecting the *E. onukii* egg. On the ten tea shoots of Longjing 43, Maoxie, Huangjinya, and Zijuan appeared green dots, their eggs hatched into 35, 32, 31 and 26 nymphs of *E. onukii,* respectively, indicating that the eggs checked by blue light the first time were truly generated by the *E. onukii* (Table 2).

### 3.4. Comparison between BLDM and SMDM in Detecting E. onukii Eggs

**Figure 2:**
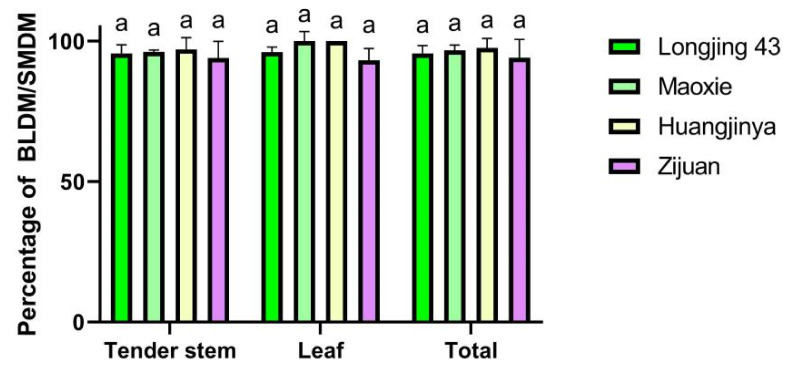
Percentage of BLDM/SMDM for the egg detection of *E. onukii* at four tea cultivars. The same letter above each column indicates no significant difference among four cultivars.

Using the BLDM and the SMDM to investigate the number of *E. onukii* eggs, there were no significant differences between these two methods in the total sum of eggs in Longjing 43 (F = 0.085, df = 5, *p* = 0.785), Maoxie (F = 2.113, df = 5, *p* = 0.220), Huangjingya (F = 0.122, df = 5, *p* = 0.745), and Zijuan (F = 1.588, df = 5, *p* = 0.276). The comprehensive proportions of BLDM/SMDM in Longjing 43, Maoxie, Huangjinya, and Zijuan were 95.61%, 96.61%, 97.38%, and 93.48%, respectively, with no significant difference between the BLDM and the SMDM (F = 1.724, df = 11, *p* = 0.239). Thus, the tea cultivar did not affect the accuracy of BLDM in detecting *E. onukii* eggs (Table 3, Figure 2). Separately, the percentages of BLDM/SMDM on the tender stems of Longjing 43, Maoxie, Huangjinya, and Zijuan were 95.55%, 96.12%, 96.94%, and 93.53%, whereas on leaves they were 96%, 100%, 100%, and 93.18%, respectively. Similarly, there were no significant differences in BLDM/SMDM among the four cultivars on the tender stem (F = 1.233, df = 11, *p* = 0.359) and leaf (F = 1.096, df = 11, *p* = 0.405).

Similar results were found in the egg calculations between the BLDM and the SMDM on the leaf positions in the four tea cultivars. However, the largest *E. onukii* eggs in each cultivar were all oviposited at the third leaf position, and the proportions of BLDM/SMDM at this site in Longjing 43, Maoxie, Huangjinya and Zijuan were 95.93%, 99.29%, 100%, and 96%, respectively (Table 3). Therefore, these results demonstrate that *E. onukii* eggs deposited at higher leaf positions on tea shoots can be as accurately detected by the simpler method of blue light as by dissection.

### 3.5. Fertilized Ovum State of E. onukii

The mating times of five *E. onukii* females occurred at 21:59, 8:35, 18:10, 20:27, and 12:18 respectively, indicating that the mating behavior of *E. onukii* can occur at any time during the day. The gestation duration was 34 h and 31 min, 28 h and 55 min, 25 h and 50 min, 36 h and 3 min, and 31 h and 42 min for each time period, respectively, revealing that the fertilized ovum state in an *E. onukii* female was about 1–2 d after the first mating. Eggs started to be oviposited in succession after the fertilized ovum state. Thus, 1~3 eggs were laid among the five tested females within the first day, while 3~7 eggs were found in the third survey, 2–3 d later (Table 4).

### 3.6. Ovipositional Rhythm and Egg Distribution by E. onukii

*E. onukii* laid eggs throughout the 24 h under laboratory conditions, wherein the periods 20:30~23:30 and 17:30~20:30 had the highest proportions of eggs with 22.12 ± 2.02% and 17.90 ± 4.91%, respectively, as compared to other periods (Table 5). Moreover, the percentage of eggs laid during these two time periods were not significantly different from each other. The percentages of eggs in other periods were (in the order): 14:30~17:30 (13.76 ± 2.53%), 11:30~14:30 (10.73 ± 0.77%), 2:30~5:30 (9.81 ± 2.40%), 8.30~11:30 (9.43 ± 2.08%), 23:30~2:30 (9.27 ± 2.32%) and 5.50~8:30 (7.50 ± 1.12%), respectively. Despite the first two time periods and the second five time periods seeming numerically different, the percentage of eggs laid in the all eight time periods were not significantly different. This result shows that, while an apparent daily peak of egg laying occurred from 17:30 to 23:30, egg laying actually occurred haphazardly throughout the day.

**Figure 3:**
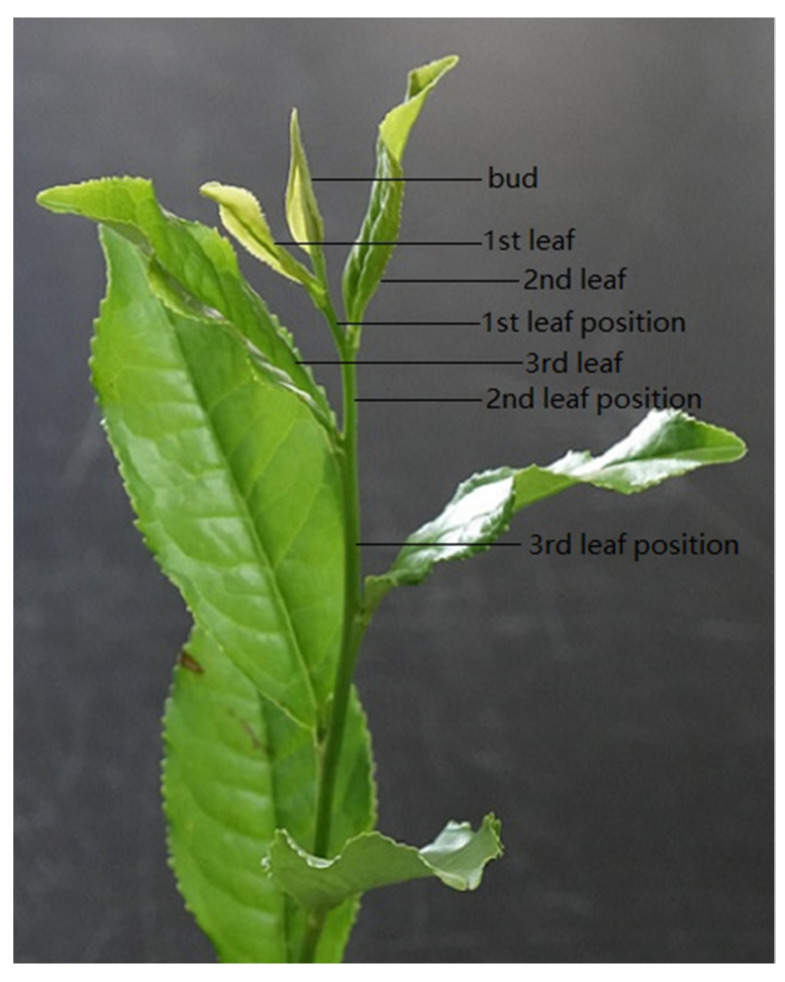
Configuration of the leaf positions on a tender tea shoot. The first leaf position was between the first and second leaf nodes, the second leaf position was between the second and third leaf nodes, the third leaf position was between the third and fourth leaf nodes, and by parity of reasoning for the other leaf positions.

**Figure 4:**
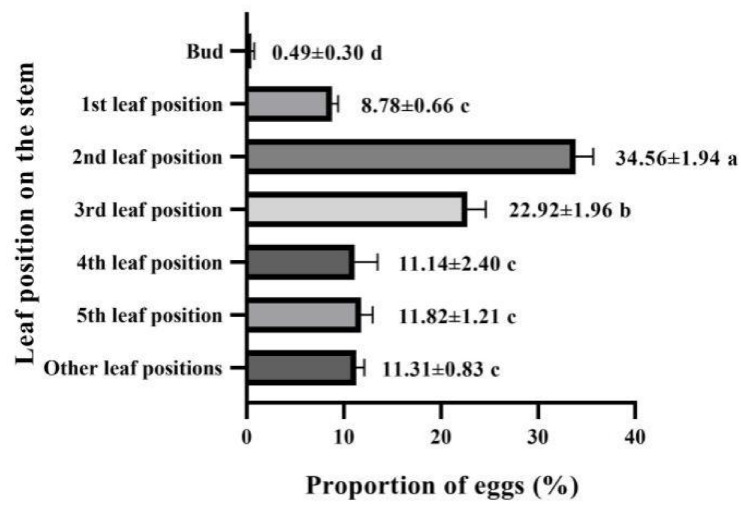
Egg distribution of *E. onukii* at different leaf positions on the tea shoots. The values (mean ± SE) with different letters in different leaf positions indicate a significant difference at the *p* < 0.05 level by LSD test.

An average proportion of 86.7% of eggs were laid by *E. onukii* females within the tender stem, while the remaining 13.3% were deposited inside leaves (included petiole and leaf veins) (Figure 3, Table 5). Egg distribution on the different leaf positions on the stem varied, with the second leaf position containing the highest percentage (34.56 ± 1.94%), which was followed by third leaf position (22.92 ± 1.96%). Both locations showed significant differences from those at other leaf positions, indicating that *E. onukii* preferred to oviposit in the second and third leaf positions on the stems. Additionally, no significant difference existed among all eggs laid in the first, fourth, fifth and other leaf positions, with the proportions of eggs 8.78 ± 0.66%, 11.14 ± 2.40%, 11.82 ± 1.21%, and 11.31 ± 0.83%, respectively, being the lowest in the bud (only 0.49 ± 0.30%; Figure 4).

### 3.7. Egg Laying by Single Females Daily and the Whole Generation

Our study demonstrated that for seven consecutive days, the average numbers of eggs laid daily by a single female of *E. onukii* were 3.10, 3.33, 2.67, and 2.51, respectively (Table 6). Furthermore, during the lifespan of an *E. onukii* adult female, the ovipositional duration was about 20 days, with the highest fertility of up to 42.8 eggs (Table 7).

## 4. Discussion

In recent decades, the tea leafhopper, *Empoasca onukii*, has become a key pest in the tea gardens of China, thus attracting attention. Due to the absence of a convenient method to detect eggs, the bionomics associated with oviposition ecology is poorly understood. Hence, the objective of this study was to identify the best detection method for tea leafhopper eggs inside tender tea tissues, in order to rapidly and accurately determine the number of eggs, and allow in-depth studies on the egg laying behavior of tea leafhopper. Leafhopper egg-detection methods have a long history. According to Goodey (1937), a staining mixture for detecting nematodes in plant tissues was developed using phenol, lactic acid, glycerin and distilled water at 1:1:2:1 proportions [24]. Thereafter, the lactophenol-acid-staining technique was gradually improved and applied to count the eggs of leafhoppers in Cicadellidae within plant tissue, such as *E. fabae* on potato and alfalfa [25,26], *E. solana* and *Amrasca biguttula* on cotton [27,28], *Nephotettix virescens* on rice [29]. Despite gradual improvements in the use of lactophenol–glycerin solution detection method, where there are associated challenges of high-cost, operating inconvenience, and low-efficiency (e.g., requiring at least 1 d for Backus’s method, and longer (up to 3 d) for Carlson and Hibbs’s method for *E. fabae,* and even 5–6 d for Khan and Saxena’s method for *Nephotettix virescens*). Additionally, the chemicals involved are hazardous and harmful to health.

The commonly used stereomicroscope detection method (SMDM) for the investigation of *E. onukii* eggs inside dissected tea shoots was time consuming, laborious, and unable to detect all the eggs. For example, very new, young eggs were difficult to locate and identify, even under the microscope, because new eggs are transparent and small in size. In addition, eggs deposited in tender shoots could sometimes be destroyed accidentally during shoot-dissecting and egg-detection under a stereomicroscope, thus reducing the accuracy of the count. Furthermore, tea shoots examined by microscopy were destructively sampled during the experiment, so follow-up studies (e.g., egg morphogenesis and hatchability investigations) could not be conducted correctly because dissected eggs cannot survive outside of shoots and rarely hatched. Therefore, with the self-fluorescence effect of *E. vitis* egg in grape leaf tissue, we evaluated the accuracy of the blue light detection method (BLDM) to assess the quantity of *E. onukii* eggs in intact tea shoots. Our results indicated that BLDM was efficient and suitable for counting *E. onukii* eggs directly in intact plant tissue. Moreover, BLDM was non-toxic, low-cost, simple to operate, produced faster output results, and was non-destructive to the tea shoots samples, compared with both the lactophenol staining and SMDMs.

Previous studies have demonstrated that *E. onukii* females gestate fertilized eggs successively and oviposit them in batches [6]. The associated relevance of our study is that *E. onukii* ovipositional characteristics can be evaluated further for their propagative behaviors. This is because we demonstrated that *E. onukii* females finished the zygote phase within 1–2 d after first mating, followed by ovipositing daily. While the ovipositional rhythm appeared to numerically peak from 17:30 to 23:30, their numbers were not significantly different from the eggs laid in other periods, suggesting that tea leafhopper can oviposit haphazardly during the whole day. From the investigations of egg deposition by 10 females for 7 consecutive days as well as five females during the whole ovipositing duration, it seemed that *E. onukii* did not have an evident rhythm for egg laying, with an average of 2–4 eggs laid daily per female. However, the highest reproductive rate was a relatively prolific 40 eggs in about 20 d. Therefore, these observations might partly explain the large numbers and widespread distribution of tea leafhoppers currently in major tea-producing areas in China [30].

Our study found that *E. onukii* largely oviposited on the tender stems of tea, with more than half of the eggs deposited at the second and third leaf positions; these results concur with previous studies [8,9,10]. Nevertheless, about one-tenth of the eggs are laid outside the tender stem, in the leaf (including petiole and vein); these non-stem eggs were often missed or ignored in previous studies. Consequently, similar comprehensive procedures should be implemented in the next studies, to better reveal the process of searching for a suitable site to insert their eggs as part of the egg laying mechanism of *E. onukii* females. Certainly, the tenderness of tea shoot tissues can be hypothesized to be a key factor contributing to oviposition preference in tea leafhopper. Thus, further studies combined with the biometric determination of tea shoot and egg laying behavior of *E. onukii* need to be conducted.

In the future, optimized strategies incorporating ovipositional manipulation methods should be developed for tea leafhopper management, based on basic research relating to the bionomics of egg laying. This article used the now-verified BLDM to test the zygote state of females, and the ovipositional rhythm, egg distribution, amount of eggs laid by a single female daily and the whole generation. Further studies are recommended concerning the egg fluorescence mechanism, ovipositional strategies, directional preference mechanisms, as well as the influence of internal and external factors on oviposition.

## 5. Conclusions

Herein, we provide the first systematic inspection and evaluation of the use of the blue light detection method (BLDM) to detect *E. onukii* eggs on the tea shoots of four tea cultivars. We have successfully demonstrated that BLDM is more convenient, effective, and precise compared to the SMDM. Further studies are recommended to assess the *E. onukii* egg laying behavior with the bionomics of oviposition.

## Figures and Tables

**Table 1:** Varietal characteristics of the four chosen tea cultivars.

Cultivar	Tree Age	Tender Stem		Leafhopper Resistance
		Thickness	Epidermis Color	
Longjing 43	5	Medium	Green	Medium
Maoxie	5	High	Green	Medium
Huangjinya	5	Medium	Golden yellow	Medium
Zijuan	5	High	Purple	High

**Table 2:** Accuracy test of BLDM for the egg detection of *E. onukii.*

Cultivar	No. of Shoots	No. of Eggs	7 d	
			No. of Hatched Nymphs	No. of Eggs
Longjing 43	10	0	0	0
	10	40	35	3
Maoxie	10	0	0	0
	10	36	32	4
Huangjinya	10	0	0	0
	10	33	31	1
Zijuan	10	0	0	0
	10	28	26	0

Blue light was used in the first detection of eggs, whereas the stereomicroscope dissection of shoots was used the second time, 7 d later. Cumulative number of hatched nymphs were counted for 7 d; thereafter, the eggs were considered still unhatched or had died because of plant defenses or parasitization by mymarid wasps. A few missed eggs might have been parasitized by Mymaridae in the field; the tiny wasps hatched out may not have been recorded during the survey.

**Table 3:** Comparison between the BLDM and the SMDM for the egg detection of *E. onukii.*

Cultivar	Detection Method	Eggs/Ten Tea Shoots								
		Bud	First Leaf Position	Second Leaf Position	Third Leaf Position	Fourth Leaf Position	Other Leaf Positions	Total on Tender Stem	Total on Leaf	Total Sum
Longjing 43	A: BLDM B: SMDM A/B (%)	2	5	20.33	55	36	41	157.33	24	181.33
		4	7.33	23.67	57.33	35.33	41	164.67	25	189.67
		50	68.18	85.92	95.93	101.89	100	95.55	96	95.61
Maoxie	A: BLDM B: SMDM A/B (%)	0.33	2	15.67	46.67	21	22	107.33	16	123.33
		0.67	4.67	16.33	47	21.67	22	111.67	16	127.67
		50	42.86	95.92	99.29	96.92	100	96.12	100	96.61
Huangjinya	A: BLDM B: SMDM A/B (%)	0.67	2.33	13	48.33	19.33	12	95	16.33	111.33
		1	4.33	13.67	48.33	19.67	12	98	16.33	114.33
		66.67	53.85	95.12	100	98.31	100	96.94	100	97.38
Zijuan	A: BLDM B: SMDM A/B (%)	1	1	17.33	24	12.67	17.33	72.33	13.67	86
		1.67	3	19.33	25	12.67	17.33	77.33	14.67	92
		60	33.33	89.66	96	100	100	93.53	93.18	93.48

Data are the mean values. BLDM: blue light detection method; SMDM: stereomicroscope detection method; A: intact tea shoots; B: dissected tea shoots.

**Table 4:** Fertilized ovum state and the initial egg laying of *E. onukii* female.

Replicate	Mating Time		First Survey (Egg in the Abdomen)			Second Survey (Egg in the Tea Shoots)			Third Survey (Egg in the Tea Shoots)		
	Date (Month/Day)	Time (h/min)	Date (Month/Day)	Time (h/min)	Zygote State (h/mins)	Date (Month/Day)	Time (h/min)	Egg	Date (Month/Day)	Time (h/min)	Egg
1	10.11	21:59	10.13	8:30	34: 31	10.14	8:30	2	10.15	13:30	4
2	10.12	8:35	10.13	13:30	28: 55	10.14	13:30	3	10.16	13:30	7
3	10.24	18:10	10.25	20:00	25: 50	10.26	8:30	1	10.28	8:30	4
4	11.12	20:27	11.14	8:30	36: 03	11.15	8:30	2	11.16	13:30	3
5	11.19	12:18	11.20	20:00	31: 42	11.21	8:30	1	11.22	13:30	3

**Table 5:** Ovipositional rhythm and egg distribution of *E. onukii.*

Period	Egg			Proportion of Egg (%, Mean ± SE)
	Tender Stem	Leaf	Total (Mean ± SE)	
2:30–5:30	11.75	1.75	13.50 ± 3.86 bc	9.81 ± 2.40
5:30–8:30	9.25	1	10.25 ± 1.93 c	7.50 ± 1.12
8:30–11:30	11.75	1.5	13.25 ± 3.50 bc	9.43 ± 2.08
11:30–14:30	12.25	2	14.25 ± 1.25 bc	10.73 ± 0.77
14:30–17:30	15.25	2.75	18.00 ± 2.94 bc	13.76 ± 2.53
17:30–20:30	17.75	4.5	22.25 ± 3.61 ab	17.90 ± 4.91
20:30–23:30	28	2	30.00 ± 4.78 a	22.12 ± 2.02
23:30–2:30	10.75	1.75	12.50 ± 3.57 bc	9.27 ± 2.32
Total	117	17	134	
Proportion (%)	86.685	13.315	-	

Data are the mean values. Different letters in the same column indicate significant difference at *p* < 0.05 level by LSD (least significant difference) test.

**Table 6:** Egg laid by a single female of *E. onukii* on a single day.

Date (Month/Day)	Eggs/Ten Females			
	Replicate 1	Replicate 2	Replicate 3	Replicate 4
8.1	14	6	17	6
8.2	30	16	10	13
8.3	42	22	16	27
8.4	34	53	35	29
8.5	18	20	20	26
8.6	45	66	40	47
8.7	34	50	49	28
Total	217	233	187	176
Mean/Female per day	3.10	3.33	2.67	2.51

**Table 7:** Total number of eggs laid by a single female of *E. onukii.*

Date (Month/Day)	Eggs/Five Females			
	Replicate 1	Replicate 2	Replicate 3	Replicate 4
8.9	40	13	23	25
8.12	38	40	10	16
8.15	20	21	10	8
8.18	30	44	22	21
8.21	46	39	23	21
8.24	31	43	4	15
8.27	9	6	3	0
8.30	0	0	0	0
Total	214	206	95	106
Mean/Female	42.8	41.2	19	21.2

## References

[1] Tang Y.C., Zhou C.L., Chen X.M. (2010). Progress in the oviposition behavioral ecology of herbivorous insects. For. Res..

[2] Cai W.Z., Pang X.F., Hua B.Z., Liang G.W., Song D.L. (2011). General Entomology.

[3] Cao X.Y., Wang Y.C., Gong X.J., Xu Z.X., Luo F. (2018). Regional occurrence characteristics of *E. (Matsumurasca) onukii* Matsuda and its integrated controlling. China Plant Prot..

[4] Qin D.Z., Zhang L., Xiao Q., Dietrich C., Matsumura M. (2015). Clarification of the identity of the tea green leafhopper based on morphological comparison between Chinese and Japanese specimens. PLoS ONE.

[5] Backus E.A., Serrano M.S., Ranger C.M. (2005). Mechanisms of hopperburn: An overview of insect taxonomy, behavior, and physiology. Annu. Rev. Entomol..

[6] Wei Q., Gao C.F. (2014). Advance in research occurrence regularity and controlling of the tea green leafhopper, *E. vitis* (Gӧthe). Tea Sci. Technol..

[7] Kosugi Y. (1996). Overwintering ecology of tea green leafhopper, *E. onukii* Matsuda in tea field. (2) First oviposition time of the overwintered female. Proc. Kansai PI. Prot..

[8] Kosugi Y. (1998). Oviposition sites of tea green leafhopper, *E. onukii* Matsuda in a new shoot of tea plants. Proc. Kansai PI. Prot..

[9] Wang Y.J., Xie Z.L., Pang X.F. (2008). Studies on the ecological niche of *E. vitis* (Göthe) and spiders in tea gardens. J. Tea Sci..

[10] Wang Q.S., Huang J., Gao X.F. (2010). Studies on the spatial distribution of *E. vitis* (Gӧthe) in organic tea garden. Chin. Agric. Sci. Bull..

[11] Chen L.L., Chen P., Wang Y., Ma X., Lin J.K., Zhao Z.H. (2019). Cover crops mediate abundance and egg density of tea green leafhopper (Hemiptera: Cicadellidae) in a tea plantation. J. Plant Prot..

[12] Li H.L., Lin N.Q. (2012). The influence of temperature and humidity on the population dynamics of small green leafhopper at tea garden. Fujian J. Agric. Sci..

[13] Li H.L., Liu F.J., Wang D.F., Zhang W.J., Wu G.Y., Lin N.Q. (2013). Effects of shading on population dynamics of small green leafhopper. Fujian J. Agric. Sci..

[14] Qiao L. (2015). Response of *E. onukii* Matsuda to Short-Term High or Low Temperature and the Molecular Mechanisms. Ph.D. Thesis.

[15] Gao M.Q. (2007). Studies on the Small Green Leafhopper, *E. vitis* (Göthe) and its Egg Parasitoids in Fujian Tea Plantations. Master’s Dissertation.

[16] Li H.L. (2008). Studies on the Bionomics of Small Green Leafhopper and its Egg Parasitoids. Master Dissertation.

[17] Xu L.Y. (2013). Effects of Intercropping Tea Plantation with Green Manure on the Green Leafhopper and Main Natural Enemies of Pests. Master’s Dissertation.

[18] Mao Y.X., Zou W., Ma X.H., Lin N.Q. (2009). Fuzzy cluster analysis of the dynamics of egg density and egg parasitism of *E. vitis* (Göthe). J. Fujian Agric. Forest. Univ..

[19] Lin J.L. (2010). Studies on Chemical and Color Communication Mechanisms among Tea and Tea Green Leafhopper and Mymarid. Master’s Dissertation.

[20] Jin S. (2012). Resistance Mechanisms of Tea Plant Cultivars to Tea Green Leafhopper. Ph.D. Thesis.

[21] Boll S., Herrmann J.V. (2001). A new method to monitor eggs of the grape leafhopper (*E. vitis*) in grapevine leaves. J. Plant Dis. Prot..

[22] Herrmann J.V., Boll S. (2004). A simplified method for monitoring eggs of the grape leafhopper (*E. vitis*) in grapevine leaves. J. Plant Dis. Prot..

[23] Kosugi Y. (1994). Effect of temperatures on development period of tea green leafhopper, *E. onukii* Matsuda. Proc. Kansai PI. Prot..

[24] Goodey T. (1937). Two methods for staining nematodes in plant tissues. J. Helminthol..

[25] Carlson O.V., Hibbs E.T. (1962). Direct counts of potato leafhopper, *E. fabae*, eggs in solanum leaves. Ann. Entomol. Soc. Am..

[26] Backus E.A., Hunter W.B., Arne C.N. (1988). Technique for staining leafhopper (Homoptera: Cicadellidae) salivary sheaths and eggs within unsectioned plant tissue. J. Econ. Entomol..

[27] Moffitt H.R., Reynolds H.T. (1972). Bionomics of *E. solana* DeLong on cotton in Southern California. Hilgardia.

[28] Singh L., Singh R. (2005). Influence of leaf vein morphology in OkraGenotypes (Malvaceae) on the oviposition of the leafhopper species *Amrasca biguttula* (Hemiptera: Cicadellidae). Entomol. Gen..

[29] Khan Z.R., Saxena R.C. (1986). Technique for locating planthopper (Homoptera: Delphacidae) and leafhopper (Homoptera: Cicadellidae) eggs in rice plants. J. Econ. Entomol..

[30] Chen Z.M. (2013). Chemical Ecology of Tea Pests.

